# Theory vs. Facts

**Published:** 1883-08

**Authors:** A. M. Ross


### THEORY vs. FACTS.

DR. A. M. ROSS.

It used to be taught that theory and practice were in opposition; that this is still quite true is without doubt, but it is also true that during the past one hundred years speculation has been making a great advance into the domain of practice. Theories developed during this period of time have been based largely upon practica- knowledge, and a property of the human mind recognized as the selecting and comparing faculty, that has been wonderfully developed. The mind is constantly receiving suggestions from the internal and the external world, and according to the capacity of the individual these suggestions are arranged, and by a process of real soning by induction or deduction certain conclusions are arrived at and disposed of, and practical theories are evolved. By a comparative inactivity of this faculty, permitting the ideas to flow spontaneously, a state of abstraction or reverie is produced, and baseless theories evolved.

Dr. Beard, in his article "Neurasthenia," Dr. Carpenter, in his "Human Physiology," Dr. Holland's "Chapters on Mental Physiology," and other authorities, very plainly show that a concentrated attention on any object of thought will intensify the impression of it, and this may proceed so far as to make ideas dominant, or the idea dominant over all else.

The individual who is constantly alert, who ever strives to cultivate this discriminative faculty, who is cautious and careful to give due weight to all sides of a question, will never be dominated by ideas, nor in the study of any problem will he be likely to run off with any of its factors as a solution of the whole.

Is there an application of this subject, of the qualities of this discriminative faculty, of this power of selecting and comparing the facts relating to dental caries, and by its application showing the domination of ideas? I think there is.

It is sensibly urged that in the study of dental caries all the facts so far developed that enter into the process should be carefully and impartially grouped; in this group order and sequence must be secured, and from it a theory of cause may be deduced. This is the logical manner in which to proceed. But is it in this way that the different theorists have advanced? If a man, by his special education can classify the proximate ingredients of the animal body, can reduce them to their ultimate elements, is able to investigate all the processes that go on in the elaboration and assimilation of new materials, the wearing out and excretion of the old, we are to infer that if he takes up the investigation of this problem of tooth decay there will be no exclusion of any of its known factors.

A glance of the educated eye at the acid theory, the inflammation theory, and the germ theory, will reveal the fact that the advocate of one theory rejects certain factors, or treats with indifference what the advocate of another makes pre-eminent in his group. But for one thing we would be at loss to understand this, because we have to suppose what is probably quite untrue, that each prepossessed investigator personally examines every factor of the problem, becoming familiar with everything co-related to it. But a survey of the field reveals the power and sway of ideas upon the subject of dental caries. As in religion, astronomy, medicine, etc., history repeats itself. The individual conceiving an idea from some external fact, elaborates a theory, consuming much after time in its defense and in the endeavor to reconcile other facts with the theory. After a time his position is found baseless and it has to be vacated. Does he acknowledge his error? Not at all. He is silent. He is, in his opinion, the friend of progress, and believes himself superior to those who prefer to study the question impartially, who view the facts in the case as pointing in no definite direction, but who believe that ultimately the cause of dental caries will be found.

It were better for us to strive after truth for the truth's sake, than to have any desire for notoriety mixed up in our endeavors. Ideas usually become associated in our minds by habit, and if we are awake, the suggestions of the external world extend a modifying influence, the will interfering and directing the train of thought. Now if the will is determined upon truth-seeking it will never be caught in the attempt to defend a weak theory. We have a noble example of this fact before us as a profession, and it would be well to follow the example and allow ourselves to become *Millerites*, to a limited degree.

